# Reply to Gomby: The utility of HOLC maps to capture historical housing discrimination

**DOI:** 10.1073/pnas.2201140119

**Published:** 2022-04-21

**Authors:** Mahasin S. Mujahid, Xing Gao, Loni P. Tabb, Colleen Morris, Tené T. Lewis

**Affiliations:** ^a^Division of Epidemiology, School of Public Health, University of California, Berkeley, CA 94720;; ^b^Department of Epidemiology and Biostatistics, Dornsife School of Public Health, Drexel University, Philadelphia, PA 19104;; ^c^Department of Epidemiology, Rollins School of Public Health, Emory University, Atlanta, GA 30322

We thank Gomby (1) for the comments regarding Home Owners’ Loan Corporation (HOLC) maps and the discriminatory history of the Federal Housing Administration (FHA). These comments provide an opportunity to clarify several points in our original paper (2), which uses HOLC maps to measure historical redlining.

Gomby's (1) assertion that redlining as a practice existed before HOLC maps is accurate, and we acknowledge that discriminatory lending practices and housing discrimination existed long before the 1930s. Our introduction states, “along with other discriminatory housing policies such as deed restrictions and racial covenants,” redlining affected Black neighborhoods (2). Although HOLC maps did not mark the beginning of redlining, they have played a critical role in institutionalizing it and housing inequities that affect health outcomes. Therefore, our research does not contradict Hillier's point that redlining reflects a pattern of lending discrimination rather than causing it (3).

We leveraged HOLC maps because they are valuable data for capturing lending discrimination. Faber (4), a spatial inequality expert, recently examined the historical roles of New Deal era programs by using census data to show higher levels of racial residential segregation in the cities and towns appraised by HOLC compared to other locations. Faber noted that, “following HOLC, FHA offered billions of dollars of housing assistance to White Americans on the condition that they did not buy homes in communities of color” (4). Thus, HOLC maps are a useful proxy because of their downstream impact. Additionally, the large geographic scale of these maps supports the usage of HOLC maps to examine population health inequities.

The studies Gomby (1) cites use localized and less available data sources (3, 5, 6), creating other epidemiologic methodological limitations acknowledged within those studies, including a lack of generalizability, exposure heterogeneity, and small sample size. Other geospatial data relevant to redlining, that is, the maps produced by the FHA, were destroyed, “a result of two lawsuits filed in federal court that alleged discrimination solely based on race on the pricing of homes constructed or sold in transitional neighborhoods in the 1950s and 1960s” (7). In other words, we are researching a topic where the best data were destroyed to cover up this racist and blatantly discriminatory practice; thus, we rely on what is, in our estimation, the next best option. We encourage future studies to find creative ways to investigate other data sources that capture the broad spectrum of housing discriminatory policies and procedures.

We conclude that “historical redlining has an enduring impact on cardiovascular risk among Black adults in the United States,” a statement that was supported by the associations we documented in our study. Regardless of whether HOLC caused redlining or is just a reflection of redlining practices, the fact remains that this discriminatory practice may have an intergenerational impact on the current-day cardiovascular health of Black people. We employed several epidemiological methods to assess the robustness of our results, including rigorous control for confounding, conducting sensitivity analyses with various outcome measures, and utilizing three different exposure assessment methods. We anticipate that future research will enhance our findings.
